# Knowledge management awareness assessment in Nigerian tertiary institutions

**DOI:** 10.12688/f1000research.18223.2

**Published:** 2019-10-16

**Authors:** Afolakemi Simbo Ogunbanwo, Julius Olatunji Okesola, Sheryl Buckley

**Affiliations:** 1 School of computing, Univesity of South Africa, Pretoria, South Africa; 2 Computational Sciences department, First Technical University, Ibadan, Nigeria

**Keywords:** Awareness level, Knowledge, knowledge management, tertiary institutions, performance

## Abstract

**Background**: Knowledge management (KM) is a recipe for increasing performance and promoting innovation in tertiary institutions. However, some scholars argue that the Nigerian educational sector is yet to fully appreciate the importance of KM as their KM awareness level is still low. Since measurement is the basic foundation to accomplish success, this paper assesses the KM awareness level in tertiary institutions of south-west Nigeria.

**Methods**: The study applied a survey method using a closed ended questionnaire administered to 50 participants from each of the 10 institutions measured by Likert scaling. Employing SPSS for data analysis, frequency count and percentage score were adopted to analyse the demographic data, and the research hypotheses were analysed with chi square test, Pearson chi square and bivariate correlation (Pearson) analysis.

**Results**: A positive relationship between awareness, current status and level of familiarity was noted. KM awareness level in the institutions is high even though there is a significant difference between the public and private universities, as well as between the students and academic staff.

**Conclusions**: Since an increase in the awareness level increases both current status and level of familiarity which often account for KM success, it is recommend that KM awareness level should continuously be improved upon in Nigerian tertiary institutions.

## Introduction

KM is a process of coordinating, organising and making institutional or organisational knowledge available for knowledge creation, sharing, storage and reuse to achieve institutional aims and objectives. Managing the existing knowledge flow in tertiary institutions is essential. According to Kayıkçı and Ozan ^1^, knowledge is a powerful tool for organisational competition and therefore becomes significant to every industry including banking, education and governmental sectors ^2– 5^. Knowledge generated should be properly managed to ensure its future availability. Therefore, tertiary institutions have moved beyond being merely a knowledge provider to students, to also curating current knowledge for future use ^6^. A number of these institutions now operate like business organisations and compete among themselves, with knowledge as their commodity. Tertiary institutions are centres for knowledge creation and sharing ^7^, and are regarded as knowledge business organizations that should devise means of gathering and disseminating knowledge for effective decision making ^8, 9^. Therefore, institutions desiring higher performance must identify, capture and circulate valuable institutional knowledge for re-use ^9, 10^.

Many studies including Demchig ^7^ and Kidwell *et al.* ^11^ have worked on the application of KM in tertiary institutions, claiming that it improves institutional capabilities in decision making and reduces the product development cycle time, as well as improving academic and administrative services. They argue that KM adoption and implementation by the institutions could result to exponential improvements in knowledge sharing, as it has a positive impact on academic research, curriculum development, student and alumni services, administrative services and strategic planning. Al-sulami, Rashid and Ali ^12^, claimed that the performance level of an institution can shoot up through the effective and efficient implementation of knowledge management. Similarly, it increases innovation giving institutions a competitive advantage over others ^13^.

KM is an emerging concept in developing countries with varying awareness and maturity levels ^14^. Charles & Nawe ^15^ discovered that staff of Mbeya University of Science and Technology (MUST) in Tanzania were not fully aware of KM practices. Demchig ^7^ conducted an assessment on level of KM maturity in Mongolian higher institutions using the Knowledge Management Capability Assessment (KMCA) model; it was revealed that maturity level of KM was in level one, indicating knowledge sharing was not discouraged in Mongolian higher institutions ^7^. Yaakub, Othman & Yousif ^16^ discovered that KM practices in Malaysian higher learning institutions is still very low, while Anvari *et al.* ^17^ found the level of KM in Firoozabad Islamic Azad University to be below average. Although KM awareness and maturity level is yet to be fully investigated amongst Nigerian tertiary institutions in the southwest geo-political zone, several Nigerian authors ^18– 20^ have found that KM is has yet to be fully implemented in Nigerian tertiary institutions.

While the literature indicates KM awareness in most developing country institutions is low ^7, 15– 17^, a number of studies do not agree ^21, 22^. Since the position is difficult to generalise due to socio-cultural differences, KM awareness in Nigerian institutions needs to be further investigated.

## Theory and Hypotheses

**Figure 1.**
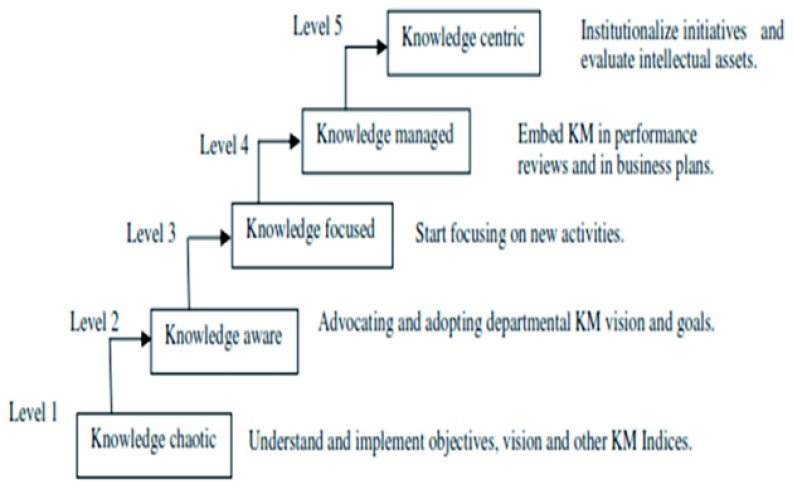
Frid”s KM Model: (adapted from Mohajah ^23^).

Assessment is regarded as “the first step towards improvement; one can’t improve what one can’t measure – formally or informally” (Kulkarni & Louis, 2003:2542). While Demchig ^7^ argued that KM current status in institution should be evaluated from the starting point. Therefore in assessing the KM status in Nigerian institutions, the study adopted Frid KM Model framework because it is simple to implement. As shown in Figure 1 below, Frid KM Model is categorized into five segments ranging from level 1 to level 5 ^23^ which are knowledge chaotic, knowledge aware, knowledge focused, knowledge managed, and knowledge centric respectively.

The Table 1 is formulated based on Frid’s KM Model as KM grading scale for measuring the state of KM in the observed institutions.

Demchig ^7^ argued that organizations with the higher level perform better in KM activities when compare with others. Hence, we formulated the following four hypotheses to test awareness levels, as well as ascertaining the difference between KM awareness levels of the public and private institutions, as well as that of the academic staff and students.

**Table 1.** KM Grading Level and Expectation.

KM Category	KM State	Expectation
Level 1	Knowledge Chaotic	Institution in this level of recognizes knowledge as an asset and has clear vision, goals and indices of KM. this stage is regarded as the starting point
Level 2	Knowledge Aware	At this level the institutions focus on adopting, developing and implementing the vision and goals of KM.
Level 3	Knowledge Focused	Institutions in this level are expected to have full implementation of level 1 and 2. Also, attention is given to KM enablers at this stage.
Level 4	Knowledge Managed	The institution embraces all the activities in level 1 to 3 and introduced KM performance review
Level 5	Knowledge Centric	The institution is expected to place emphases on establishing successful initiatives and value intellectual asset.

**Hypothesis _1_:** KM awareness level in universities in the southwest of Nigeria is high.

**Hypothesis _2_:** There is significant difference in the KM awareness level of the academic staff and student.

**Hypothesis _3_:** There is significant difference in the KM awareness level between public and private institutions in southwest Nigeria.

**Hypothesis _4_:** There is a relationship between awareness, current status and KM familiarity in the tertiary institution in in the southwest of Nigeria.

## Methods

This section discusses appropriate sampling methods employed as well as the instrumentations adopted, and reported following the Strengthening the Reporting of Observational Studies in Epidemiology (STROBE) reporting guidelines ^24^.

### Sampling method

This study adopted both probability and non-probability sampling to examine the awareness level of KM in Nigerian tertiary institution. The research population frame is the 46 accredited universities in south west Nigeria while the total population is 550 comprising both academic staff and students of selected tertiary institutions in South West Nigeria. Stratified random sampling was adopted to select 11 universities out of 46 accredited universities only. The stratified random sampling used is as follows:

- Firstly the population was grouped into three stratums - federal, state and private containing 7, 11, and 28 universities respectively.
- Secondly, systematic random sampling was used to select item from each stratum.
- Lastly, the size of each stratum was kept proportional to the sizes of the strata thereby resulting in picking two federal, three state and six private universities.

Purposive sampling was used to select the names of the 11 universities involved in the research from each of the stratum, as well as the participants consisting of academic staff and students from the selected universities. The student population outnumbers staff in every university therefore, the authors decided to gather a sample of students to staff at a ratio of 3:2. To avoid data overload and have a manageable sample size, a total number of 50 respondents (30 students and 20 members of academic staff) were selected from each university to arrive at 550 (50x11) sample size.

The questionnaire (see Extended data ^25^) was personally administered to the 10 universities involved as one university backed out from the research. The 10 universities involved in the research were five public (two federal, three state) and five private. A total number of 50 respondents were selected from each university and 500 questionnaires were administered out of which only 456 were returned and used for the analysis. The Ethical Committee of the University of South Africa issued an authorization memo to approve the questionnaire.

### Instrumentation

Likert scaling was adopted to measure awareness levels in each institution. Questions on level of KM awareness were assigned a score 1 to 4 for ‘none’, ‘low’, ‘high’ and ‘very high’ respectively while questions on knowledge recognition were respectively tagged with score 1 to 4 for ‘strongly disagree’, ‘disagree’, ‘agree’ and ‘strongly agree’. Similarly, questions on current status were assigned score tags of 1 to 4 for ‘not in existence’, ‘on pipeline’, developing’ and ‘matured’ respectively, while the level of familiarity were assigned a score ranging from 1 to 4 for ’unaware’, ‘introductory’, ‘intermediate’ and ‘advance’ respectively.

 To test the reliability of the research instrument, Cronbach’s alpha reliability test was conducted generating a result of 0.845, thereby confirming the consistency, reliability and acceptability of the factors used. Similarly, the questionnaire was pre-tested using two institutions different from those involved in the study. Administering 60 questionnaires on 30 participants from each institutions, responses and comments obtained helped to identify and address potential hitches prior to performing the actual research.

### Ethical consideration

In line with UNISA research ethics policy, all participants had the study explained to them before their recruitment. All participants provided written informed consent to participate. 

### Data analysis

IBM Statistical Programme for Social Sciences version 21 was adopted for this data analysis. Descriptive statistics of frequency counts and percentage scores was employed to analyse the demographic data, while the participants’ responses were analysed using percentage count. Hypothesis 1 was analysed with one sample chi square test, hypotheses 2 and 3 were by Pearson chi square, and hypothesis 4 was by Spearman’s rho – a non-parametric correlations.

For both the chi square and Pearson correlation coefficient, a p value <0.05 (5% significant) as ruled below.

Rule 1       If the p value is greater than 0.05 (p<0.05) accept the null hypothesis
Rule 2       If the p value is less than 0.05 (p>0.05) accept the alternate hypothesis
Rule 3       0.00<R<0.33 indicates weak relationship
Rule 4       0.34<R<0.66 indicates moderate relationship
Rule 5       0.67<R<1.0 indicates strong relationship

## Result

### Demographic characteristics of the respondents

Table 2 shows the demographic characteristics of the respondents. The total number of returned questionnaires was 456, 82% of the 500 questionnaires administered. Of these, 55% were male while 45% were female. Regarding the academic qualification of the respondent, the majority respondents were undergraduates (63%), follow by those with a master’s degree (19%), PhDs (9%) and Bachelor’s degree (9%). In terms of respondents’ status, the majority were students (67%), with academic staff making up 33% of the sample. Public universities constituted 55% while private universities made up 45% ( Table 2 and Underlying data ^26^).

**Table 2.** Demographic characteristics of the respondents.

		Frequency	Percentage
**Gender**	Male	250	55
	Female	206	45
**Qualification**	Undergraduate	288	63
	Bachelor’s Degree	40	9
	Master’s Degree	87	19
	PhD	41	9
**Status**	Student	304	67
	Staff	152	33
**Institution**	Public	250	55
	Private	206	45

### Test of research hypotheses

To test the KM awareness level in the sampled institutions, a one-sample chi square test was applied to hypothesis 1. KM awareness levels were defined as ‘none’, ‘low’, ‘high’ and ‘very high’ ( Table 3). The expected N for all the variables was 114. The results show that the hypothesis was accepted with test statistics value 295.930 ^a^, expected count of 114 and p value of 0.001.

**Table 3.** Awareness levels.

	Observed N	Expected N	Residual
None	2	114.0	-112.0
Low	116	114.0	2.0
High	256	114.0	142.0
Very high	82	114.0	-32.0
Total	456		

To test for possible differences in KM awareness level between academic staff and students, a one-sample chi square test was also used to test the hypothesis 2. The hypothesis was accepted as the test statistics value obtained was 24.794, the expected count was 0.64 and p value of 0.001.

In terms of differences in KM awareness levels between public and private institutions, the outcome of the one sample chi square test on hypothesis 3 confirms the acceptance of the alternate hypothesis with chi square test value of 10.301, expected count 0.90 and p value 0.016. 

Pearson correlation was applied on hypothesis 4 to determine the correlation between the KM awareness level, KM current status and KM familiarity. The hypothesis was accepted as the p value was 0.001. The result as depicted on Table 4 shows that there is a moderate relationship between the variables (KM awareness level, KM current status and KM familiarity) with a correlation coefficient range (r) of 0.35 < |r| < 0.45.

**Table 4.** Correlations ^b^ Matrix.

		KM Awareness Level	KM Current Status	KM Familiarity
KM Awareness Level	Pearson Correlation	1	0.450 **	0.359 **
	Sig. (2-tailed)		0.000	0.000
KM Current Status	Pearson Correlation	0.450 **	1	0.414 **
	Sig. (2-tailed)	0.000		0.000
KM Familiarity	Pearson Correlation	0.359 **	0.414 **	1
	Sig. (2-tailed)	0.000	0.000	

KM – knowledge management **. Correlation is significant at the 0.01 level (2-tailed). b. Listwise N=456

## Discussion and conclusion

Although KM implementation is important to tertiary institutions ^7, 11– 13^, assessment must come before implementation ^19, 27^. This study was therefore conducted in the context of the current literature, the majority of which suggests that KM is still emerging in developing countries ^7, 16– 19, 28^, and yet to be fully implemented in Nigeria ^18– 20^.

The study investigated knowledge management awareness in Nigerian South-west tertiary institutions and addressed the relationship between awareness, familiarity and current status of KM level. It was discovered that there is significant difference in KM awareness level amongst the public and private universities. Awareness levels between academic staff and students is also significantly different, conforming with the findings of Krubu and Krub ^29^ and Akuegwu and Nwiue ^30^ where heads of department were more involved in KM practice. This study also empirically provides evidence for correlation between the awareness, familiarity and current status of KM level. Also. It was found that there is a positive relationship between awareness, current status and level of familiarity. This suggests that if awareness levels increases, more people/institutions will practice KM and its current status will improve thereby shifting the state from developing to maturing. Similarly, KM awareness levels in south west tertiary institution was found to be high, confirming the previous studies of Ohiorenoya and Eboreime ^31^ and Oke, Ogunsemi and Adeeko ^32^. However, since KM awareness level in both the public and private institutions in the South West region in Nigeria is also high and in level 2 as specified by Frid’s KM model, this study concludes that Nigerian institutions recognise the importance of KM towards achieving institutional innovations and higher performance.

Further research may be needed to investigate the level of KM maturity and the relationship between KM and academic performance in Nigerian institutions.

## Data availability

### Underlying data

Figshare: Knowledge Management Awareness. https://doi.org/10.6084/m9.figshare.7730480.v1 ^26^

This project contains the following underlying data:

- knowledge_management_F1000.sav (Study participants knowledge management awareness data)

### Extended data

Figshare: Knowledge management awareness questionnaire. https://doi.org/10.6084/m9.figshare.7764644.v1 ^25^

This project contains the following extended data:

- F1000_Questionnaire_KM_awareness.docx (Study questionnaire)

Data are available under the terms of the Creative Commons Attribution 4.0 International license (CC-BY 4.0).
