# Supplementary material for: Knowledge management awareness assessment in Nigerian tertiary institutions
Source: F1000Res. 2019 Oct 16;8:608. Originally published 2019 May 1. [Version 2] doi: 10.12688/f1000research.18223.2 (PMC6816449; doi:10.12688/f1000research.18223.2)
Supplement: Peer review history

# Peer review: Reviewer response for version 2

**DOI:** 10.5256/f1000research.23109.r55240

**Published:** 2019-10-23

**Authors:** Magiswary Dorasamy

**Affiliations:** 1 Faculty of Management, Multimedia University, Cyberjaya, Malaysia

Author has addressed all corrections. The paper is now improved.

I confirm that I have read this submission and believe that I have an appropriate level of expertise to confirm that it is of an acceptable scientific standard.

# Peer review: Reviewer response for version 1

**DOI:** 10.5256/f1000research.19935.r53536

**Published:** 2019-09-24

**Authors:** Magiswary Dorasamy

**Affiliations:** 1 Faculty of Management, Multimedia University, Cyberjaya, Malaysia

The paper investigated KM awareness among tertiary level institutions. Respondents were academics. Data were collected appropriately. However, the paper seems to be missing theoretical framework. A strong theory will be good to lead the direction of the hypotheses. Any of the KM models would be applied for this studies to enhance its contribution in the context of Nigerian tertiary education. Some unique constructs may play important role in the awareness level of KM in Nigeria. A detail lit review may help to determine this.

I confirm that I have read this submission and believe that I have an appropriate level of expertise to confirm that it is of an acceptable scientific standard, however I have significant reservations, as outlined above.

# Peer review: Reviewer response for version 1

**DOI:** 10.5256/f1000research.19935.r47958

**Published:** 2019-05-30

**Authors:** Aderonke Oni

**Affiliations:** 1 Department of Computer and Information Sciences, Covenant University, Ota, Nigeria

The object of the study is clear and the research method was clearly outlined as well. However, the following should be attended to:

- The authors should work of the grammatical structure. Personal pronoun e.g. "we" should be avoided.
- The authors should add more significant recommendations.
- Full meaning of "STROBE" should be written before abbreviation.

I confirm that I have read this submission and believe that I have an appropriate level of expertise to confirm that it is of an acceptable scientific standard.
